# Ecology and abundance of a relict population of the bush cricket *Saga pedo* in the Northern Apennines, Italy

**DOI:** 10.1002/ece3.11381

**Published:** 2024-05-20

**Authors:** Emanuele Repetto, Pietro Milanesi, Livia De Caria, Francesca Della Rocca

**Affiliations:** ^1^ Department of Biosciences University of Milan Milan Italy; ^2^ Department of Biology and Biotechnology “L. Spallanzani” University of Pavia Pavia Italy; ^3^ Department of Biological, Geological and Environmental Sciences University of Bologna Bologna Italy

**Keywords:** abundance estimation, Bayesian framework, detection probability, habitat directive, N‐mixture models, orthoptera

## Abstract

The expansion of forest cover and intensification of agriculture represent the main threats to the bush cricket *Saga pedo*, currently listed as Vulnerable globally by the IUCN and included in Annex IV of the European Union Habitats Directive. Gathering information on its ecology and population size is challenging due to its low abundance and localized distribution. Additionally, the elusive and cryptic behavior of this species reduces the likelihood of its detection, potentially resulting in population underestimations. Thus, in this study, we aimed to (1) estimate *S. pedo* population size in relation to environmental variables and prey availability and (2) predict abundance of *S. pedo* in our study area for future monitoring in nearby territories. We found that the population of *S. pedo* in our study area consists of 197 (±115) individuals with a detection probability of 21.01% (±11.09). Detection probability of *S. pedo* further decreases on windy days. Moreover, we found that the investigated population of *S. pedo* occupies suboptimal areas, as highlighted not only by the predicted abundances but also by the association between *S. pedo* and other subfamilies of orthoptera that are ecologically very distant from our target species and mostly linked to mesophilic biotopes. Most of the individuals we observed are concentrated in small clearings completely within wooded matrices and therefore isolated from each other. Based on our results, it is possible that forest expansion toward open meadows represents the main threat to this population, transforming the clearings and xeric meadows (to which *S. pedo* is linked) into small and fragmented patches that are suboptimal and insufficient to host viable populations.

## Graphical abstract

Our study reveals that the expansion of forest cover and intensification of agriculture have led to the decline of Saga pedo, a species currently listed as Vulnerable. The population of Saga pedo in the study area is estimated to be 197 individuals, but detection is challenging due to the species' elusive behavior and the impact of environmental conditions such as wind. Our study suggests that forest expansion poses a threat to our target species, as it transforms their preferred habitats into small, fragmented, and suboptimal patches.

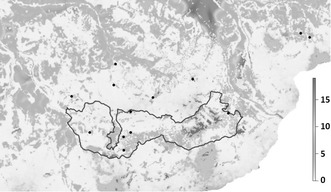

## 1 INTRODUCTION

Seminatural grassland habitats are essential ecosystems for biodiversity conservation. The high diversity of animal and plants found in this habitat depends largely on traditional agro‐pastoral practices, which limit the competition among plants and promote the coexistence of different plant species and favoring the surviving of many rare and sensitive insect species (Della Rocca et al., 2023; Sochera et al., 2013). However, seminatural grasslands are in sharp decline in Europe (Bullock et al., 2011) due to their transformation into intensive crops or the abandonment of agro‐pastoral activities, which favors natural succession and forest invasion (Queiroz et al., 2014). This process has negative effects on species richness and the composition of communities (Della Rocca et al., 2023) leading to local extinctions and a reduction in the overall genetic diversity of populations, further exacerbating the impacts of habitat loss and fragmentation. Among the species that live in seminatural grasslands, the predatory bush cricket, *Saga pedo* (Pallas, 1771; *S. pedo*, hereafter), is a priority species for conservation.

*Saga pedo* is one of the largest insects in Europe (Krištin & Kaňuch, 2007) and can reach up to 75 mm in body length and almost 120 mm when including the ovipositor. Among the 17 species of the genus *Saga* distributed across Europe and Asia (Cigliano et al., 2018), *S. pedo* is the only one occurring in Italy (Massa et al., 2012). *S. pedo* is highly sensitive to environmental changes due to its biological and ecological characteristics (Krištin & Kaňuch, 2007). It is a parthenogenetic species and its reproductive rate is low, with egg hatching between two and seven years post‐deposition (Adžić et al., 2023; Lemonnier‐Darcemont et al., 2016). Furthermore, its survival depends on high availability of preys, primarily Orthoptera and Mantodea, with a preference for large Ensifera (Castiglione et al., 2019) while its habitat is characterized by dry, open areas with sparse vegetation. These habitats range from forest steppes and limestone grikes to abandoned vineyards and slopes with southwestern or western aspects. It also thrives in areas with steppe‐like vegetation featuring open or semi‐open herbaceous‐shrub zones (Anselmo, 2019). The species' pronounced thermophily and heliophily lead it to concentrate in xerothermic patches (Galvagni & Prosser, 2004; Magistretti & Ruffo, 1959; Vergari et al., 2017), making it a primary insect indicator of these habitats (Massa et al., 2016). Thus, *S. pedo* has been significantly impacted by the expansion of forests due to the abandonment of agropastoral activities such as mowing and extensive grazing (Wilson et al., 2012). This, coupled with significant agricultural intensification and urbanization, has led to a dramatic decrease in seminatural grasslands. Moreover, the use of pesticides has further contributed to the reduction of *S. pedo*'s prey, thereby indirectly impacting the species' survival (Ancillotto & Labadessa, 2023). Pesticides can also directly impact *S. pedo* (Krištin & Kaňuch, 2007). This is further evidenced by the species' distribution in other EU countries, where its occurrence is limited to habitats with low pesticide use, even in seminatural or agricultural areas such as vineyards (Adžić et al., 2023; Anselmo, 2019; Lemonnier‐Darcemont et al., 2016). Despite having a wide Ponto‐Mediterranean distribution, which ranges from the Iberian Peninsula to western Siberia and China, this species is characterized by strongly localized and isolated populations (Krištin & Kaňuch, 2007). In the past, it likely had a more uniform distribution (Kaltenbach, 1970). In Italy, it is rare (Fontana & Cussigh, 1996; Galvagni & Prosser, 2004; Massa et al., 2012), with only a few known locations in the Northern Apennine (Anselmo, 2019; Maioglio & Repetto, 2022; Sindaco et al., 2012).

For all the above‐mentioned reasons, *S. pedo* is currently listed on the Annex IV of the Habitats Directive and Appendix II of the Bern Convention. Classifying the conservation status of *S. pedo* populations through monitoring activities and maintaining viable populations of this species is an obligation derived from Article 11 and Article 17 of the Habitat Directive (Council Directive 92/43/EEC). Indeed, the conservation of relict populations is vital for the species' survival and the preservation of its genetic variability (Habel & Schmitt, 2014). This is particularly true for species like *S. pedo*, which have highly fragmented ranges (Rösch et al., 2015). However, due to the low abundance and strong localization of its populations, collecting information on the ecology of *S. pedo* and its current population sizes is challenging (Holuša et al., 2013), especially because, as a highly threatened species, census techniques involving the capture and marking of individuals are not recommended (Nachman & Skovgård, 2012).

Thus, understanding the ecology of *S. pedo* studying its habitats is crucial to foster effective conservation strategies for this species. However, to date, ecological studies on *S. pedo* are limited (Adžić et al., 2023; Anselmo, 2019, 2022; Holuša et al., 2013) and, due to the strong elusiveness and crypticity of this species, the likelihood of observing it in nature is extremely low, resulting in underestimation of population abundance (Holuša et al., 2013).

Thus, in this study, we aimed at (1) deriving accurate estimates of *S. pedo* abundance in relation to environmental characteristics and predict it across our entire study area to identify unsampled sites for future monitoring efforts and conservation, (2) quantify the relationship and association between *S. pedo* and its potential preys at the sampling sites.

To achieve our goals, we developed a set of N‐mixture models (Royle, 2004), related abundances of *S. pedo* with those of its potential prey at sampling sites, and finally, derived a Bray–Curtis similarity index to analyze the spatial association between *S. pedo* and its potential prey.

## 2 MATERIALS AND METHODS

### 2.1 Study area

**FIGURE 1:**
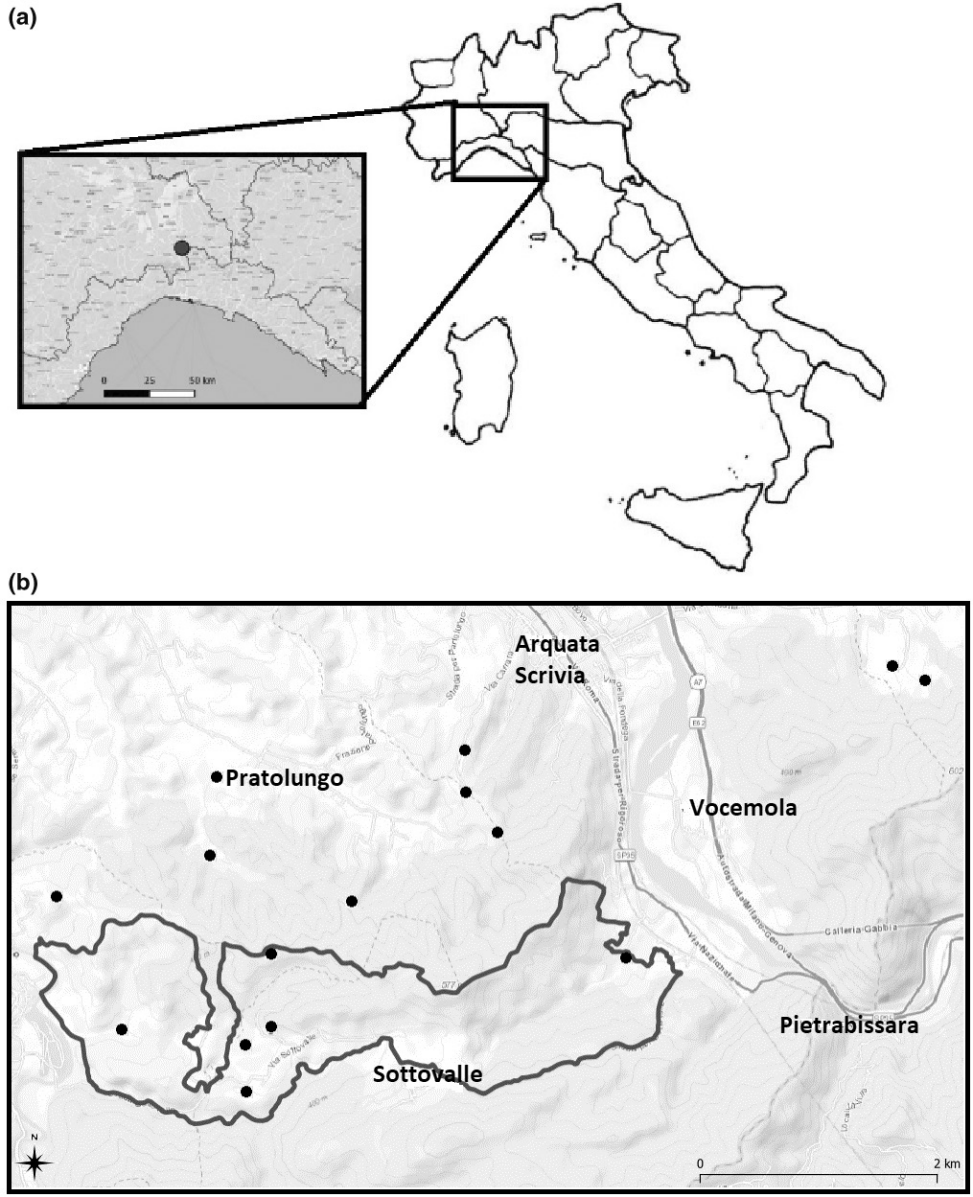
Study area: (a) location of our study area in Italy (black dot); (b) distribution of the 16 sampling sites within our study area (black dots) and borders of the Sites of Community Importance “Calanchi di Rigoroso, Sottovalle e Carrosio” (delimited by black lines).

This research was carried out in a hilly region in the Province of Alessandria, specifically within the municipalities of Arquata Scrivia, Grondona, Gavi, and Carrosio, covering an area of approximately 60 km^2^ (Figure 1a). The study area encompasses the Site of Community Importance (SCI) IT1180030, known as “Calanchi di Rigoroso, Sottovalle e Carrosio.” This SCI stretches in an east–west direction across the initial foothills of the Apennines, within the territories of the municipalities of Arquata Scrivia and Carrosio. Our study area is characterized by an heterogeneous, fragmented landscape, marked by significant badlands formations of landscape interest. Broad‐leaved forests dominate the environment, covering about half of the land (Rossi et al., 2021). The primary forest types include mesoxerophilous oak forests (*Quercus pubescens*), hornbeam‐elm forests, many of which are invasive, and chestnut groves. Other notable formations include broom shrublands (*Spartium junceum*) and xeric meadows. These environments are under threat because of forest expansion due to the cease of mowing, similar to abandoned agricultural and cultivated areas, which are mainly characterized by improved grasslands (Rossi et al., 2021). Within these open areas, dry meadows have been identified, specifically Habitat type (NATURA 2000 Code) 6210 and 6210* (important orchid sites), which are of community interest due to the abundance of orchids, including rare species (Vai, 2020). Our study area hosts other habitats of interest, some associated with aquatic environments, such as hygrophilous formations of calcifying mosses (Cratoneurion: Habitat type NATURA 2000 Code 7220*), and alluvial forests populated by black alder, white alder, and white willow (Habitat type NATURA 2000 Code 91E0*) (Vai, 2020). Of particular interest to our study are the xeric meadow environments, largely attributable to Habitat type 6210 and 6210*, where *S. pedo* completes its entire life cycle (Anselmo, 2019). In these meadows, rare plant species occur, including *Himantoglossum adriaticum*, an orchid listed in Annexes II and IV of the Habitat Directive, along with 20 Orchidaceae species (Vai, 2020). Among the arthropods, several species listed in the Habitat Directive occur: *Zerynthia cassandra* (Rossi et al., 2021), Annex IV; *Eriogaster catax*, Annexes II and IV; *Euplagia quadripunctaria*, Annex II; *Lucanus cervus*; *Austropotamobius pallipes*, Annexes II and IV; and *S. pedo* (Maioglio & Repetto, 2022). Additionally, *Oxygastra curtisii*, listed in Annexes II and IV, has been reported in the nearby area on the biodiversity platform GBIF (https://doi.org/10.15468/dl.g87qax; accessed on 21st January 2024). Within the orthopteran fauna, 70 species have been identified in our study area (Sindaco et al., 2012).

### 2.2 Data collection and covariates of detection and abundance

This study was carried out over a period of 3 years (2021–2023) during the spring/summer season, from the first week of April until the second half of August. Within our study area, *S. pedo* was monitored by observing individuals along 53 walking transects, which were included in 16 sampling sites (Figure 1b). Eight of these sites were located within the Site of Community Importance (SCI) IT1180030 “Calanchi di Rigoroso, Sottovalle e Carrosio,”, while the other eight outside the SCI but in its proximity. The distances between sampling sites varied from 150 to 7600 m, and each site was isolated from its closest sites by physical barriers such as gullies, hills, roads, and streams, or by ecological barriers to *S. pedo* like forests and strips of unsuitable habitat.

We carried out three to four 10‐m‐length transect walks with a width of 2 m at each sampling site, once a week by a single operator. Similar to Massa et al. (2016) and Anselmo (2019), individuals were sought during the walking transect using a drop‐down sweep net with a diameter of 35 cm and by inspecting shrubby vegetation with the aid of a metal pole/stick. Each individual identified was immediately recorded and released to minimize stress on the animals and avoid duplicate counts. All other orthopterans observed along the transect were also classified. These specimens were collected, preserved in 70% ethyl alcohol, and transported to the laboratory for identification. All the specimens collected were identified with the help of the expert Paolo Fontana (see Acknowledgments) and following the nomenclature of “Fauna d'Italia” (Massa et al., 2012). For the most of these specimens, we were able to classify up to the subfamily level, and for some specimens, we were able to identify the species level.

To estimate *S. pedo* detection probability, we also measured temperature and relative humidity at the beginning, middle, and end of each transect, together with the time of the day (minutes) in which surveys were carried out (Table 1). Additionally, we assessed wind intensity on a scale from 1 to 3, where 1 = total absence of wind, 3 = wind capable of compromising the use of the sweep net (Table 1).

**TABLE 1:** Covariates considered in the development of N‐mixture models to estimate abundance and detection probability of *Saga pedo* in our study area. Mean, standard deviation (SD), minimum, median, and maximum values are shown.

Covariates	Unit	Mean (±SD)	Minimum	Median	Maximum
*Abundance*					
Altitude	m a.s.l.	331.06 ± 47.33	251.69	333.72	401.71
Slope	°	14.91 ± 4.68	8.53	14.42	22.58
Tree cover density	n/m^2^	27.43 ± 14.91	0.87	25.21	53.11
Open meadows	%	11.22 ± 19.18	0	0.04	59.86
Shrublands	%	1.32 ± 3.77	0	0	13.79
Woodlands	%	39.38 ± 34.3	0	38.82	91.46
Human settlements	%	1.58 ± 1.82	0	0.58	4.57
Distance to croplands	m	51.99 ± 129.85	0	0	405.17
Distance to wetlands	m	609.07 ± 320.22	176.21	583.11	1392.79
Distance to mowed meadows	m	77.11 ± 148.43	0	0	485.01
Distance to ummowed meadows	m	269.74 ± 333.78	0	122.11	963.29
Distance to shrublands	m	195.66 ± 229.97	0	139.81	688.01
Distance to dry meadows	m	142.25 ± 206.81	0	0	664.03
Detection					
Time	min	745.19 ± 170.36	420.09	749.11	1136.01
Temperature	°C	21.91 ± 5.87	6.46	22.26	30.61
Temperature_SD	°C	1.38 ± 0.94	0.15	1.14	5.36
Humidity	%	74.77 ± 18.78	41.29	72.85	100
Humidity_SD	%	8.48 ± 6.51	0	8.34	27.21
Wind	Classes	1.18 ± 0.49	1	1	3

To estimate *S. pedo* abundance, we initially considered a set of 13 covariates (Table 1) ecologically relevant for our target species (Anselmo, 2022). Specifically, we derived two topographic covariates from the TINITALY 1.1 dataset at 10 m resolution (https://tinitaly.pi.ingv.it/Download_Area1_1.html), tree cover density from the Copernicus Tree Cover Density 2018 dataset at 10 m resolution (https://land.copernicus.eu/en/products/high‐resolution‐layer‐tree‐cover‐density/tree‐cover‐density‐2018), while lad cover covariates from “Land cover Piemonte (2022)” vector dataset (https://www.geoportale.piemonte.it/cms/progetti/land‐cover‐piemonte). All the above‐mentioned covariates were resampled at a spatial resolution of 100 m.

### 2.3 Statistical analysis

To estimate the abundance of *S. pedo*, we developed N‐mixture models with Poisson error distribution (function “pcount” in the R package “Unmarked”; Fiske & Chandler, 2011). These models offer significant advantages over traditional ones, such as estimating species abundance avoiding individual marking (Royle Repeated Count model‐RRC; Royle, 2004), which significantly decreases individuals' mortality due to handling (Nachman & Skovgård, 2012). Moreover, taking into account imperfect detection, estimated from data collected during multiple surveys at the same sites (i.e., repeated visits), N‐mixture models reduce the risk of type II errors (false negatives or underestimations of counts; MacKenzie et al., 2006), enhancing model performance and providing robust estimates of species abundance (Della Rocca et al., 2020; Fiske & Chandler, 2011; Nachman & Skovgård, 2012; Royle, 2004; Royle & Nichols, 2003). Specifically, we adopted the information‐theoretic approach with multimodel inference (function “dredge” in the R package “MuMIn”; v.1.0.0. Barton, 2009). This method involves generating as many models (N‐mixture, in our case) as potential combinations of the covariates considered (listed in Table 1), except to those including correlated covariates (where |*r*| > .7; Dormann et al., 2012). The resulting models were ranked using the corrected Akaike criterion (AICc, Akaike Information Criterion; Akaike, 1973). Specifically, we chose the model with the lowest AICc as the “best”' model and then ranked the remaining models as the difference (ΔAICc) between the AICc of the best model and those of the other models. Additionally, for each model, we computed the “Akaike weight” (wi), which can be interpreted as the probability that a given model is the best among all models considered (Akaike, 1981). Following the recommendations of Anderson and Burnham (2002), we considered only models with ΔAICc < 2 (including the best model, ΔAICc = 0).

To test for relations between the abundance of *S. pedo* and those of individuals of each orthopteran subfamily (potential preys) at the sampled sites, we conducted GLMs. Also in this case, we adopted the Information‐Theoretic Approach with multimodel inference (function “dredge” in the R package “MuMIn”; v.1.0.0. Barton, 2009). In this analysis, we alternately considered as dependent variable the number of *S. pedo* observations collected (1) during each transect and (2) during each sampling session. The predictive variables included (1) the count of individuals from each orthopteran subfamily present concurrently with *S. pedo* in the same transects and sessions; (2) the total abundance of orthopterans; and (3) the total number (16) of subfamilies observed concurrently with *S. pedo* in each transect and for each sampling session.

To visually represent the relationship between *S. pedo* and the orthopteran subfamilies in a spatial context, we employed the Principal Coordinate Analysis (PCO) metric ordering method (Gower, 1966) using the PERMANOVA+ add‐on package for PRIMER v6 (Anderson et al., 2008). In this method, points plotted on the graph are arranged based on similarity measures. These measures are calculated using Bray–Curtis similarity index (Bray & Curtis, 1957) derived from the abundance data of orthopteran subfamilies collected on transects. Spearman rank correlation of |*r*| > .7 was used to show relationships between individual subfamilies and PCO axes. The abundance data for orthopteran subfamilies were transformed using the logarithmic function Log (*X* + 1).

## 3 RESULTS

**FIGURE 2:**
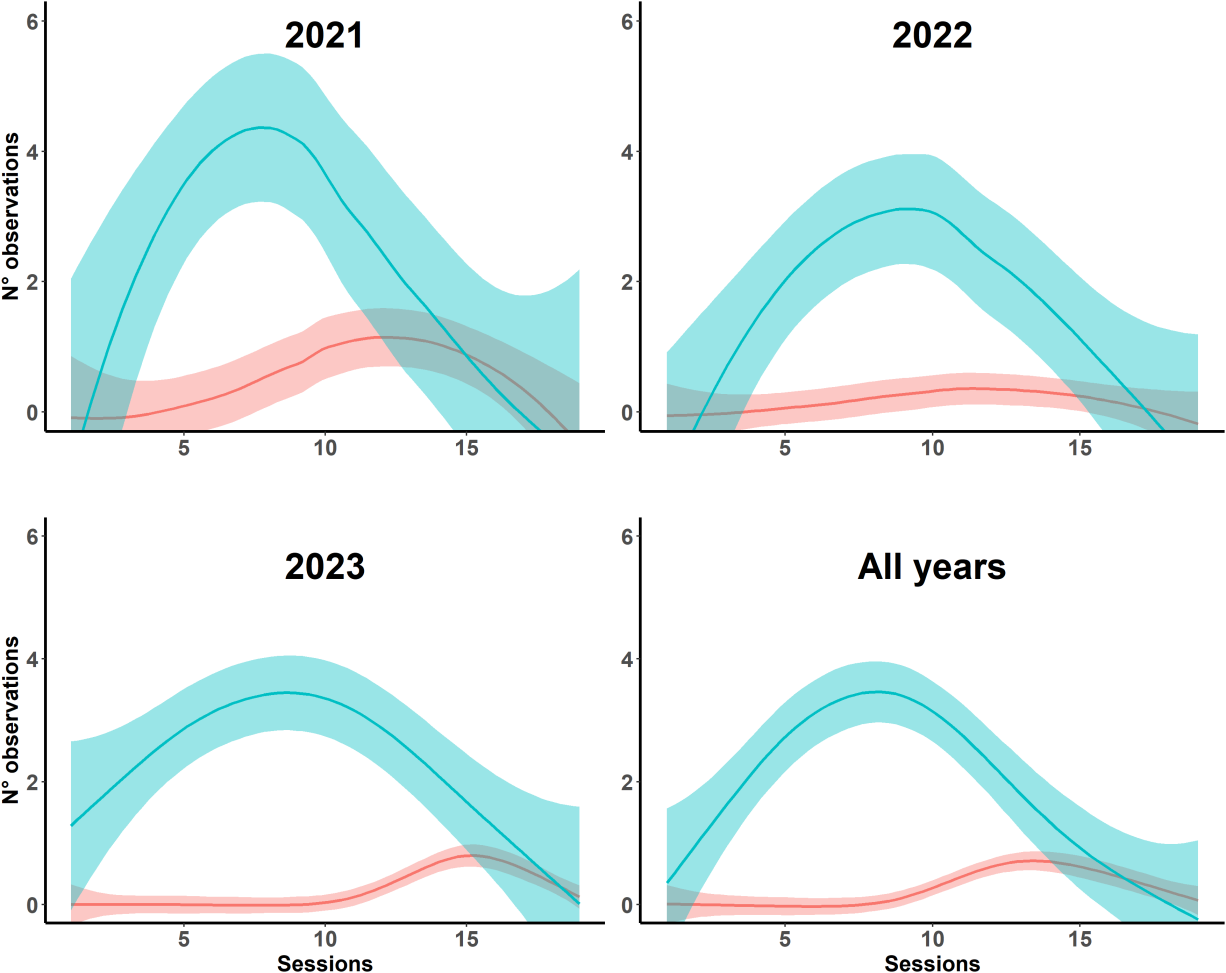
Number of observations of bush cricket in relation to sampling sessions, for the years 2021–2023 and average of these 3 years. Juveniles are shown in light blue while adults in red (±95% confidence intervals are also shown).

Throughout the entire sampling period, *S. pedo* was observed 337 times in 12 out of 16 sampling sites (Table S1) with the first sighting of the season on April 14 and the last on August 5. Almost half of these observations (185 in total) were made at a single sampling site located outside SCI (Site A1, Table S1). At this site, up to 13 individuals were observed in a single day. In the sampling sites located within the SCI, we observed *S. pedo* 117 times, with a maximum of six individuals observed in a single day at a single sampling site (Site B1, Table S1). In all the other sites investigated, the occurrence of our target species was sporadic and inconsistent (Table S1). Most of the individuals, primarily juveniles, were observed between the second week of May and the second week of June. After this period, the majority of the observed individuals were adults, although they were seen less frequently (Figure 2).

**FIGURE 3:**
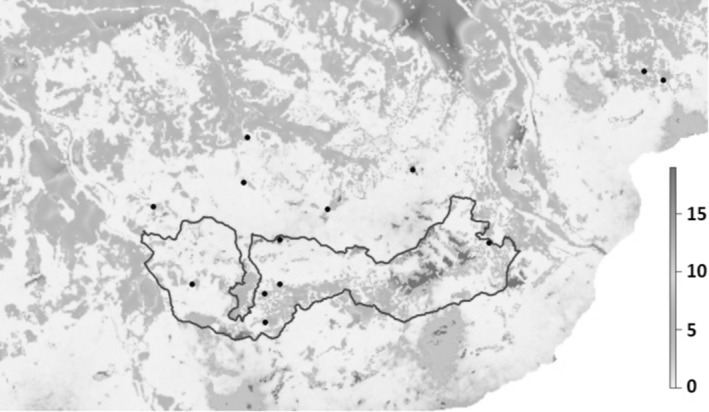
Predicted abundance of *Saga pedo* in our study area through multimodel inference, using N‐mixture models.

Through multimodel inference, we identified and then averaged a total of seven N‐mixture models with ΔAICc < 2, showing the environmental variables that most significantly relate to the abundance of *S. pedo* while estimating its detection probability, 21.01% (±11.09). The likelihood of observing the species during the sampling session was higher during the afternoon and evening hours and lower on more humid days (Table 2). Thus, we estimated a population abundance of 197 individuals (±115) of *S. pedo*, directly related to slope, the percentage of open meadows, and the distance to shrublands. On the other hand, the abundance of *S. pedo* was negatively influenced, albeit to a lesser extent, by the distance to unmowed meadows (Table 2). Thus, based on these variables, we projected *S. pedo* abundance over our study area (Figure 3).

**TABLE 2:** Average estimates of the standardized coefficients (*β*), standard errors (SE), *p*‐values (*p*), and relative importance from Akaike weights (*W*) of the covariates entered in the seven best models (ΔAICc < 2) to estimate Saga pedo abundance through multimodel inference, using N‐mixture models.

Covariates	*β*	SE	*p*	*W*
*Abundance*				
Intercept	2.79	0.37	<.0001	–
Slope	1.25	0.83	<.05	24.99
Open meadows	0.79	0.08	<.05	24.99
Distance to shrublands	0.61	0.23	<.05	0.16
Distance to ummowed meadows	−0.64	0.47	<.05	0.024
*Detection probability*				
Intercept	−2.81	0.37	<.0001	–
Time	0.21	0.09	<.0001	24.99
Humidity	−0.41	0.16	<.0001	24.99

We collected 3500 specimens, including *S. pedo* belonging to 40 species, 17 subfamilies, and 5 families (Acrididae, Gryllidae, Mogoplistinae, Tettigoniidae, Tetrigidae; Table S2). All specimens were classified at least to the subfamily level, 2872 up to the genus level, and of these, 1795 at the species level.

Through multimodel inference between *S. pedo* and orthopteran abundances, we identified 17 models with ΔAICc < 2 (Table 3). These models encompass all 18 variables considered for the analyses. Our results showed a direct and significant relation between the abundance of *S. pedo* and the richness of subfamilies occurring in our study area (Table 3). No other significant relations were identified.

**TABLE 3:** Average estimates of the standardized coefficients (*β*), standard errors (SE), *p*‐values (*p*), and relative importance from Akaike weights (*W*) of the covariates (i.e., Orthoptera subfamilies abundances) entered in the 17 best models (ΔAICc < 2) to estimate *Saga pedo* abundance through multimodel inference approach, using generalized linear models.

Covariates	*β*	SE	*p*	*W*
(Intercept)	−2.386	67.325	.97	–
Catantopinae	2.809	494.447	1	9.182
Gomphocerinae	23.819	4006.359	1	9.182
N° of individuals	−53.281	8773.897	1	9.182
N° of subfamilies	0.738	0.29	<.0001	9.182
Calliptaminae	18.546	2890.958	.99	8.696
Oedipodinae	2.19	349.082	1	8.696
Phaneropterinae	13.871	2195.427	.99	8.696
Tettigoniinae	23.263	3610.005	.99	8.696
Gryllomorphinae	1.004	108.715	.99	7.556
Acridinae	1.306	190.83	.99	4.865
Oecanthinae	2.293	358.23	.99	4.563
Tetriginae	0.944	108.342	.99	3.549
Mogoplistinae	−3.829	341.052	.99	3.017
Cyrtancathacridinae	−2.568	294.775	.99	2.404
Bradyporinae	1.502	198.654	.99	1.399
Malanoplinae	−1.114	310.808	1	0.763
Nemobiinae	−0.784	96.164	.99	0.37

**FIGURE 4:**
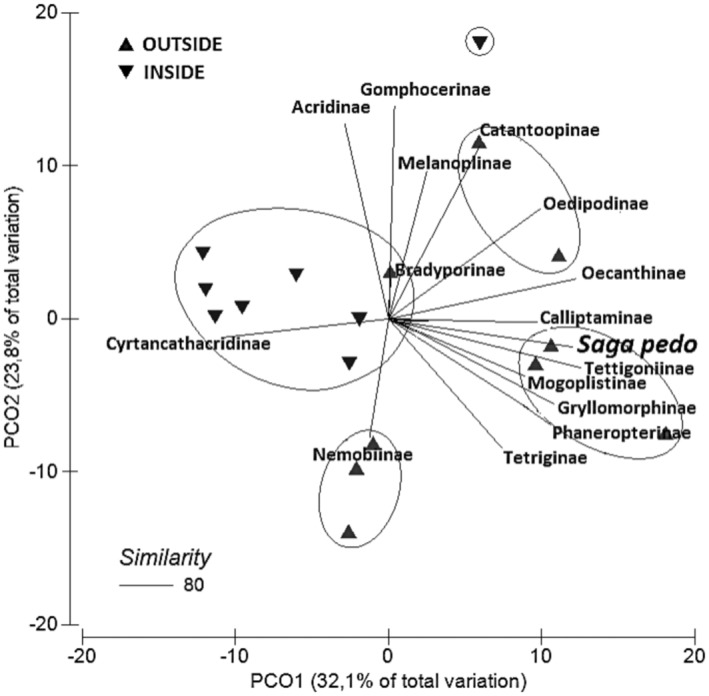
Scatter plot showing four clusters derived by principal coordinate analysis (PCO) carried out on Bray–Curtis distance matrix between sampling sites (black triangles) with a similarity degree of 80%: one group is located inside the Site of Community Importance (SCI) “Calanchi di Rigoroso Sottovalle e Carroso” while the other three outside (*Saga pedo* belongs to one of these three clusters, along with the subfamilies Tettigoniinae, Calliptaminae, Mogoplistinae, Gryllomorphinae, and Phaneropterinae). Correlation of taxa toward sampling sites are indicated in the length and direction of vectors.

However, PCO reveals that *S. pedo* preferred areas used by other subfamilies, particularly Tettigoniinae, Calliptaminae, Mogoplistinae, Gryllomorphinae, and Phaneropterinae (Figure 4). Moreover, PCO indicated a clear separation in terms of similarity in the composition of orthopterans between the sites inside the SCI “Calanchi di Rigoroso, Sottovalle e Carrosio” and those outside the SCI (accounting for 32.1% of the variance). We also found that *S. pedo* is more abundant in sites outside the SCI.

## 4 DISCUSSION

We found that the investigated population of *S. pedo* appears to be well‐established in our study area, occupying almost all the sampling sites, although with a fragmented distribution and often limited to small relic meadows. During our data collection, the likelihood of encountering the species was notably low on average, especially when compared to other endangered insects listed in the annexes of the Habitat Directive. For example, the stag beetles *Lucanus cervus* have a detection probability of 56.2% on transects (Della Rocca et al., 2020), while the larvae of *Parnassius apollo* have a detection probability ranging from 84% to 97% (Fred & Brommer, 2003; Kukkonen, 2021). The low detectability of *S. pedo* has been previously documented in the literature (Anselmo, 2022; Holuša et al., 2013; Iorio et al., 2019). However, this is the first study that, through extensive repeated samplings and advanced statistical models (N‐mixture models), provides robust estimates of the detection probability of *S. pedo* without the need for handling insects. Research on detection probability for orthopterans is limited, often stemming from monitoring conducted through song listening, which has proven to be highly effective (Penone et al., 2013). However, this sampling method is not applicable to *S. pedo* due to the absence of males and song production. However, the detection probability estimated with such methods for other orthopterans ranged from 43% to 98% (McNeil & Grozinger, 2020), significantly higher than that for *S. pedo*. The low detectability of *S. pedo* should be interpreted in light of the species' ecological characteristics. Indeed, this species is not only cryptic and associated with dense vegetation but is also less active during the hottest hours of the day (Lemonnier‐Darcemont et al., 2016) leading to a reduction of detectability to very low abundances (Pellet et al., 2012). Additionally, as already reported by other researchers and also according to our results, *S. pedo* has particularly low population densities in its later stages and among adults (Holuša et al., 2013). While we carried out a relatively large number of (repeated) surveys at each sampling sites (*N* = 20), we see the possibility to optimize the sampling effort to five‐ten surveys, similar to the approach of Bröder et al. (2020), which aims to identify the minimum number of sampling occasions needed to obtain unbiased and robust estimates of population size of elusive insect species. Surveys of *S. pedo* should be scheduled from the middle of May to the middle of June for juveniles and from the middle of June to the middle of July for adults, according to the peak of observations during our sampling sessions.

Most individuals of *S. pedo* found in the study area are concentrated within small, isolated xeric grasslands, surrounded by a predominantly wooded matrix. Based on the results of the N‐mixture models developed in this study that indicate a clear correlation between *S. pedo* and the open and unmanaged grasslands, it is evident that the current presence of the species in the investigated territory is to be considered suboptimal for the species. Considering the forest expansion since the 1950s in the Northern Apennines (Malandra et al., 2019), it is probable that many habitats suitable for *S. pedo* have been replaced by forests. Furthermore, the vast arid meadows have likely experienced significant fragmentation. The disappearance and fragmentation of meadows and clearings are among the major threats to *S. pedo* conservation (Adžić et al., 2023; Anselmo, 2019; Holuša et al., 2013; Krištin & Kaňuch, 2007; Lemonnier‐Darcemont et al., 2016), especially considering its limited mobility. Individual movement abilities of *S. pedo* range between 0.5 and 37.5 m traveled per day, and adult individuals did not traverse even very small wooded areas, not reaching suitable sites just 10 m away (Holuša et al., 2013). This low vagility combined with a parthenogenetic reproduction makes our target species particularly sensitive to fragmentation and prone to isolation. From this perspective, even a narrow strip of dense forest could act as a barrier to dispersal.

Our study revealed a shorter phenology of the species in 2021 and 2022, lasting only 70 days, compared to the 144 days documented by Schall (2002) and the 164 days observed in captivity by Lemonnier‐Darcemont et al. (2016). This could be attributed to unfavorable environmental factors and the low density of adult individuals, which may reduce their detectability due to dispersal (Krištin & Kaňuch, 2007). On the contrary, in 2023, the duration of the life cycle (112 days) was similar to that reported in literature (Lemonnier‐Darcemont et al., 2016; Schall, 2002). However, we noticed an earlier disappearance of adults (late July‐early August) while they are usually found until September or October (Massa et al., 2012).

We found higher abundances of *S. pedo* in areas with a higher diversity of its potential prey. However, the subfamilies where it was most commonly found displayed ecological characteristics that, to some extent, diverge from those of *S. pedo*. For instance, some subfamilies such as Mogoplistinae, represented by *Arachnocephalus vestitus*, inhabit ecotonal, arid environments between forests and meadows (Iorio et al., 2019; Massa et al., 2012). Others, like Gryllomorphinae, represented by *Gryllomorpha dalmatina*, are associated with litter and hypogean fresh environments (Iorio et al., 2019; Massa et al., 2012). Tettigoniinae and Phaneropteriinae are also abundant, with both subfamilies consisting of species adapted to a wide range of environmental conditions (Iorio et al., 2019; Massa et al., 2012). The environmental heterogeneity of the investigated territory is also confirmed by the simultaneous presence of xerophilic species such as Antaxius (Chopardius) pedestris, which are associated with densely shrub‐covered areas (Dreux, 1962; Galvagni, 2001), and mesophilic species such as Leptophyes laticauda, which are linked to herbaceous edges, bushy pastures, forest edges, and clearings (Buzzetti et al., 2010). In our study, we also were able to confirm the occurrence of species such as *Acrida ungarica mediterranea* and *Omocestus raymondi*, whose distribution in our study area was previously unknown or uncertain (De Caria et al., 2023).

The issue of forest expansion, leading to the disappearance of grasslands and shrublands, is prevalent in many regions where *S. pedo* exists. Various studies, including one by Holuša et al. (2013), have proposed solutions such as extensive grazing and tree vegetation cutting to maintain open habitats. These methods are seen as potential and favorable options in our study area.

Given the high fragmentation of the population under study and the forest acting as a significant barrier to individual exchange, it would be beneficial to carry out these interventions in areas connecting the subpopulations under investigation. This would ensure enhanced genetic flow.

Simultaneously, it would be advantageous to manage abandoned meadows and pastures, or those lacking agricultural upkeep and not yet overtaken by invasive forest vegetation. Management should be based on criteria that promote the preservation of habitat characteristics and prevent the neglect and alteration of the floral composition.

## 5 CONCLUSIONS

The conservation of *S. pedo*, a parthenogenetic species with limited dispersal capabilities, is indeed urgent and crucial. The fragmentation of its habitat and the dwindling population size pose significant risks to its survival. Efforts to preserve this species are vital, especially in regions like Piedmont where known populations are sparse. The presence of Natura 2000 sites offers a beacon of hope, as these protected areas can serve as critical refuges for *S. pedo* and other species that share its habitat. Our study could indeed act as a catalyst for increased awareness and the development of effective management strategies. These strategies should aim to maintain habitat connectivity, which is essential for the movement and genetic diversity of species. By enhancing the grasslands within these sites, not only is the future of *S. pedo* safeguarded, but also the ecological integrity of the entire area, benefiting a multitude of species that rely on these habitats for their survival. It's a multifaceted approach that requires collaboration between scientists, conservationists, and policymakers to ensure the successful implementation of these conservation efforts.

## AUTHOR CONTRIBUTIONS

**Emanuele Repetto:** Conceptualization (equal); data curation (lead); investigation (lead); methodology (lead); writing – original draft (equal); writing – review and editing (equal). **Pietro Milanesi:** Formal analysis (equal); methodology (equal); software (equal); writing – review and editing (equal). **Livia De Caria:** Data curation (equal); investigation (equal). **Francesca Della Rocca:** Conceptualization (lead); data curation (equal); formal analysis (equal); investigation (equal); methodology (equal); project administration (lead); supervision (lead); writing – original draft (lead); writing – review and editing (equal).

## CONFLICT OF INTEREST STATEMENT

The authors declare no conflicts of interest.

## Supporting information


Table S1

## Data Availability

Species data collected in this study are available in Table S1.
