# Supplementary material for: Ecology and abundance of a relict population of the bush cricket Saga pedo in the Northern Apennines, Italy
Source: Ecol Evol. 2024 May 20;14(5):e11381. doi: 10.1002/ece3.11381 (PMC11103639; doi:10.1002/ece3.11381)
Supplement: Supplementary file 1 — Table S1 [file ECE3-14-e11381-s001.docx]

| Sampling days (SD) | N obs. | Samplig sites (SS) | | | | | | | | | | | | | | | |  |
| --- | --- | --- | --- | --- | --- | --- | --- | --- | --- | --- | --- | --- | --- | --- | --- | --- | --- | --- |
|  |  | **A1** | **A2** | **A3** | **B1** | **B2** | **B3** | **B5** | **B6** | **B7** | **B8** | **C1** | **C2** | **C3** | **C4** | **D1** | **D2** | **Tot. SS** |
| I-Apr. | Tot | 0 | 0 | 0 | 0 | 0 | 0 | 0 | 0 | 0 | 0 | 0 | 0 | 0 | 0 | 0 | 0 | 0 |
|  | Max | 0 | 0 | 0 | 0 | 0 | 0 | 0 | 0 | 0 | 0 | 0 | 0 | 0 | 0 | 0 | 0 |  |
| II-Apr. | Tot | 0 | 0 | 0 | 2 | 0 | 0 | 0 | 1 | 0 | 1 | 0 | 0 | 0 | 0 | 0 | 0 | 4 |
|  | Max | 0 | 0 | 0 | 2 | 0 | 0 | 0 | 1 | 0 | 1 | 0 | 0 | 0 | 0 | 0 | 0 |  |
| III-Apr. | Tot | 2 | 0 | 0 | 2 | 0 | 1 | 0 | 2 | 0 | 0 | 2 | 1 | 0 | 0 | 0 | 0 | 10 |
|  | Max | 2 | 0 | 0 | 2 | 0 | 1 | 0 | 2 | 0 | 0 | 2 | 1 | 0 | 0 | 0 | 0 |  |
| IV-Apr. | Tot | 1 | 0 | 0 | 4 | 0 | 0 | 0 | 0 | 0 | 0 | 3 | 0 | 0 | 0 | 0 | 0 | 8 |
|  | Max | 1 | 0 | 0 | 4 | 0 | 0 | 0 | 0 | 0 | 0 | 3 | 0 | 0 | 0 | 0 | 0 |  |
| I-May | Tot | 9 | 1 | 0 | 3 | 0 | 0 | 1 | 0 | 0 | 1 | 0 | 0 | 0 | 0 | 0 | 0 | 15 |
|  | Max | 8 | 1 | 0 | 2 | 0 | 0 | 1 | 0 | 0 | 1 | 0 | 0 | 0 | 0 | 0 | 0 |  |
| II-May | Tot | 31 | 1 | 0 | 7 | 0 | 3 | 1 | 0 | 1 | 0 | 0 | 0 | 0 | 0 | 0 | 0 | 44 |
|  | Max | 13 | 1 | 0 | 6 | 0 | 3 | 1 | 0 | 1 | 0 | 0 | 0 | 0 | 0 | 0 | 0 |  |
| III-May | Tot | 10 | 0 | 1 | 3 | 0 | 0 | 1 | 0 | 0 | 2 | 0 | 0 | 0 | 0 | 0 | 0 | 17 |
|  | Max | 4 | 0 | 1 | 2 | 0 | 0 | 1 | 0 | 0 | 2 | 0 | 0 | 0 | 0 | 0 | 0 |  |
| IV-May | Tot | 20 | 2 | 0 | 4 | 0 | 1 | 0 | 0 | 3 | 1 | 4 | 1 | 0 | 0 | 0 | 0 | 36 |
|  | Max | 6 | 1 | 0 | 3 | 0 | 1 | 0 | 0 | 3 | 1 | 4 | 1 | 0 | 0 | 0 | 0 |  |
| I-June | Tot | 29 | 0 | 1 | 7 | 1 | 4 | 0 | 0 | 0 | 4 | 2 | 0 | 0 | 0 | 0 | 0 | 48 |
|  | Max | 10 | 0 | 1 | 4 | 1 | 4 | 0 | 0 | 0 | 4 | 2 | 0 | 0 | 0 | 0 | 0 |  |
| II-June | Tot | 25 | 5 | 1 | 9 | 1 | 3 | 1 | 0 | 0 | 0 | 0 | 0 | 0 | 0 | 0 | 0 | 45 |
|  | Max | 9 | 4 | 1 | 4 | 1 | 3 | 1 | 0 | 0 | 0 | 0 | 0 | 0 | 0 | 0 | 0 |  |
| III-June | Tot | 13 | 1 | 2 | 6 | 1 | 0 | 3 | 0 | 3 | 3 | 0 | 0 | 0 | 0 | 0 | 0 | 32 |
|  | Max | 6 | 1 | 2 | 3 | 1 | 0 | 3 | 0 | 3 | 3 | 0 | 0 | 0 | 0 | 0 | 0 |  |
| IV-June | Tot | 23 | 1 | 0 | 5 | 0 | 1 | 0 | 0 | 0 | 1 | 0 | 0 | 0 | 0 | 0 | 0 | 31 |
|  | Max | 11 | 1 | 0 | 1 | 0 | 1 | 0 | 0 | 0 | 1 | 0 | 0 | 0 | 0 | 0 | 0 |  |
| I-July | Tot | 12 | 2 | 2 | 6 | 0 | 1 | 0 | 0 | 0 | 2 | 0 | 0 | 0 | 0 | 0 | 0 | 25 |
|  | Max | 4 | 1 | 2 | 2 | 0 | 1 | 0 | 0 | 0 | 2 | 0 | 0 | 0 | 0 | 0 | 0 |  |
| II-July | Tot | 4 | 1 | 0 | 1 | 0 | 0 | 0 | 0 | 0 | 3 | 1 | 0 | 0 | 0 | 0 | 0 | 10 |
|  | Max | 3 | 1 | 0 | 1 | 0 | 0 | 0 | 0 | 0 | 3 | 1 | 0 | 0 | 0 | 0 | 0 |  |
| III-July | Tot | 2 | 0 | 1 | 0 | 0 | 0 | 0 | 0 | 0 | 0 | 0 | 0 | 0 | 0 | 0 | 0 | 3 |
|  | Max | 2 | 0 | 1 | 0 | 0 | 0 | 0 | 0 | 0 | 0 | 0 | 0 | 0 | 0 | 0 | 0 |  |
| IV-July | Tot | 1 | 0 | 0 | 0 | 0 | 0 | 1 | 0 | 0 | 0 | 0 | 0 | 0 | 0 | 0 | 0 | 2 |
|  | Max | 1 | 0 | 0 | 0 | 0 | 0 | 1 | 0 | 0 | 0 | 0 | 0 | 0 | 0 | 0 | 0 |  |
| V-July | Tot | 2 | 0 | 0 | 1 | 0 | 0 | 0 | 0 | 0 | 1 | 0 | 0 | 0 | 0 | 0 | 0 | 4 |
|  | Max | 2 | 0 | 0 | 1 | 0 | 0 | 0 | 0 | 0 | 1 | 0 | 0 | 0 | 0 | 0 | 0 |  |
| I-Aug. | Tot | 1 | 0 | 0 | 0 | 0 | 2 | 0 | 0 | 0 | 0 | 0 | 0 | 0 | 0 | 0 | 0 | 3 |
|  | Max | 1 | 0 | 0 | 0 | 0 | 2 | 0 | 0 | 0 | 0 | 0 | 0 | 0 | 0 | 0 | 0 |  |
| II-Aug. | Tot | 0 | 0 | 0 | 0 | 0 | 0 | 0 | 0 | 0 | 0 | 0 | 0 | 0 | 0 | 0 | 0 | 0 |
|  | Max | 0 | 0 | 0 | 0 | 0 | 0 | 0 | 0 | 0 | 0 | 0 | 0 | 0 | 0 | 0 | 0 |  |
| II-Aug. | Tot | 0 | 0 | 0 | 0 | 0 | 0 | 0 | 0 | 0 | 0 | 0 | 0 | 0 | 0 | 0 | 0 | 0 |
|  | Max | 0 | 0 | 0 | 0 | 0 | 0 | 0 | 0 | 0 | 0 | 0 | 0 | 0 | 0 | 0 | 0 |  |
| Tot. SD | | 185 | 14 | 8 | 60 | 3 | 16 | 8 | 3 | 7 | 19 | 12 | 2 | 0 | 0 | 0 | 0 | **337** |

Table S1. The table shows data from a three-year study (2021-2023) on the frequency of *Saga pedo* sightings. It includes the total count of observations (Tot) made over the three years, as well as the maximum number of specimens seen in a single sampling day (Max) at each site. Sampling were performed once a week for 20 weeks each year.

| **Family** | **Subfamily** | **Species** | **Juv** | **Sub** | **Ad** | **Tot** |
| --- | --- | --- | --- | --- | --- | --- |
| Acrididae | Acridinae | *Acrida ungarica mediterranea* Dirsh, 1949 | 16 | 0 | 0 | 16 |
| Acrididae | Calliptaminae | *Calliptamus barbarus barbarus* (Costa, 1836) | 0 | 0 | 6 | 6 |
| Acrididae | Calliptaminae | *Calliptamus italicus italicus* (Linnaeus, 1758) | 0 | 0 | 6 | 6 |
| Acrididae | Calliptaminae | *Calliptamus siciliae* Ramme, 1927 | 0 | 0 | 19 | 19 |
| Acrididae | Calliptaminae | *Calliptamus sp.* | 582 | 34 | 0 | 616 |
| Acrididae | Catantopinae | *Pezotettix giornae* (Rossi, 1794) | 64 | 5 | 0 | 89 |
| Acrididae | Cyrtancathacridinae | *Anacridium aegyptium* (Linnaeus, 1764) | 5 | 0 | 0 | 5 |
| Acrididae | Gomphocerinae | *Chorthippus (Glyptobothrus) brunneus brunneus* (Thunberg, 1815) | 1 | 0 | 9 | 10 |
| Acrididae | Gomphocerinae | *Chorthippus (Chorthippus) dorsatus dorsatus* (Zetterstedt, 1821) | 1 | 0 | 3 | 4 |
| Acrididae | Gomphocerinae | *Euchorthippus declivus* (Brisout de Barneville, 1848) | 290 | 15 | 53 | 358 |
| Acrididae | Gomphocerinae | *Euthystira brachyptera* (Ocskay, 1826) | 7 | 10 | 10 | 27 |
| Acrididae | Gomphocerinae | *Omocestus (Omocestus) raymondi* (Yersin, 1863) | 0 | 0 | 1 | 1 |
| Acrididae | Gomphocerinae | *Omocestus (Omocestus) rufipes* (Zetterstedt, 1821) | 9 | 13 | 30 | 52 |
| Acrididae | Gomphocerinae | *Omocestus (Omocestus) uvarovi* Zanon, 1926 | 0 | 0 | 1 | 1 |
| Acrididae | Gomphocerinae | *Omocestus sp* | 1 | 0 | 0 | 1 |
| Acrididae | Gomphocerinae | Undetermined specimens | 547 | 29 | 0 | 576 |
| Acrididae | Melanoplinae | *Odontopodisma decipens insubrica* Nadis, 1980 | 3 | 0 | 0 | 3 |
| Acrididae | Oedipodinae | Undetermined specimens | 51 | 0 | 0 | 51 |
| Gryllidae | Gryllinae | *Eumodicogryllus bordigalensis* (Latreille, 1804) | 1 | 0 | 0 | 1 |
| Gryllidae | Gryllomorphinae | *Gryllomorpha (Gryllomorpha) dalmatina* (Ocskay, 1832) | 0 | 0 | 1 | 1 |
| Gryllidae | Nemobiinae | *Nemobius sylvestris sylvestris* (Bosc, 1792) | 1 | 0 | 0 | 1 |
| Gryllidae | Oecanthinae | *Oecanthus dulcisonans* Gorochov, 1993 | 0 | 1 | 0 | 1 |
| Gryllidae | Oecanthinae | *Oecanthus pellucens pellucens* (Scopoli, 1763) | 4 | 2 | 0 | 6 |
| Gryllidae | Oecanthinae | *Oecanthus* cfr. *dulcisonans/pellucens* | 29 | 0 | 0 | 29 |
| Mogoplistidae | Mogoplistinae | *Arachnocephalus vestitus* (Costa, 1855) | 4 | 5 | 1 | 10 |
| Tetrigidae | Tetriginae | *Tetrix depressa* Brisout de Barneville, 1848 | 0 | 0 | 1 | 1 |
| Tetrigidae | Tetriginae | *Tetrix sp.* | 2 | 0 | 0 | 2 |
| Tettigonidae | Bradyporinae | *Ephippiger perforatus* (Rossius, 1790) | 4 | 0 | 0 | 4 |
| Tettigonidae | Phaneropterinae | *Leptophyes laticauda* (Frivaldsky, 1867) | 5 | 7 | 2 | 14 |
| Tettigonidae | Phaneropterinae | *Leptophyes punctatissima* (Bosc, 1792) | 2 | 0 | 0 | 2 |
| Tettigonidae | Phaneropterinae | *Metaplastes pulchripennis* (Costa, 1863) | 22 | 1 | 0 | 23 |
| Tettigonidae | Phaneropterinae | *Tylopsis lilifolia* (Fabricius, 1793) | 255 | 31 | 36 | 322 |
| Tettigonidae | Phaneropterinae | *Leptophyes* cfr. *laticauda/punctatissima* | 273 | 3 | 0 | 276 |
| Tettigonidae | Phaneropterinae | *Phaneroptera* cfr. *falcata/nana* | 37 | 0 | 0 | 37 |
| Tettigonidae | Saginae | *Saga pedo* (Pallas, 1771) | 50 | 5 | 3 | 58 |
| Tettigonidae | Tettigoniinae | *Antaxius (Chopardius) pedestris* (Fabricius, 1787) | 63 | 7 | 0 | 70 |
| Tettigonidae | Tettigoniinae | *Decticus albifrons* (Fabricius, 1775) | 9 | 1 | 1 | 11 |
| Tettigonidae | Tettigoniinae | *Decticus verrucivorus verrucivorus* (Linnaeus, 1758) | 6 | 0 | 0 | 6 |
| Tettigonidae | Tettigoniinae | *Eupholidoptera chabrieri* (Charpentier, 1825) | 60 | 0 | 0 | 60 |
| Tettigonidae | Tettigoniinae | *Pholidoptera fallax* (Fischer, 1853) | 1 | 1 | 3 | 5 |
| Tettigonidae | Tettigoniinae | *Platycleis grisea* (Fabricius, 1781) | 0 | 0 | 2 | 2 |
| Tettigonidae | Tettigoniinae | *Platycleis romana* Ramme, 1927 | 0 | 2 | 8 | 10 |
| Tettigonidae | Tettigoniinae | *Rhacocleis neglecta* (Costa, 1863) | 4 | 3 | 2 | 9 |
| Tettigonidae | Tettigoniinae | *Roeseliana azami minor* Nadig, 1961 | 2 | 0 | 0 | 2 |
| Tettigonidae | Tettigoniinae | *Sepiana sepium* (Yersin, 1854) | 129 | 8 | 3 | 140 |
| Tettigonidae | Tettigoniinae | *Tessellana tessellata tessellata* (Charpentier, 1825) | 0 | 0 | 1 | 1 |
| Tettigonidae | Tettigoniinae | *Tettigonia viridissima* (Linnaeus, 1758) | 80 | 13 | 7 | 100 |
| Tettigonidae | Tettigoniinae | *Yersinella beybienkoi* La Greca, 1974 | 190 | 19 | 1 | 210 |
| Tettigonidae | Tettigoniinae | *Yersinella raymondii* (Yersin, 1860) | 125 | 2 | 1 | 128 |
| Tettigonidae | Tettigoniinae | *Decticus* cfr. *albifrons/verrucivorus* | 20 | 0 | 0 | 20 |
| Tettigonidae | Tettigoniinae | *Platycleis* cfr. *grisea/romana* | 32 | 0 | 0 | 32 |
| Tettigonidae | Tettigoniinae | *Rhacocleis* cfr. *germanica/neglecta* | 35 | 0 | 0 | 35 |
| Tettigonidae | Tettigoniinae | *Yersinella* cfr. *beybienkoi/raymondi* | 13 | 1 | 0 | 14 |
| Tettigonidae | Tettigoniinae | *Pholidoptera sp.* | 16 | 0 | 0 | 16 |
| **Total** |  |  | **3071** | **218** | **211** | **3500** |

**Table S2.** List of orthopteran species, with the relative number of specimens (**Juv**: Number of juveniles; **Sub**: Number of subadults; **Ad**: Number of Adults; **Tot**: Total number of specimens), collected in the dry grasslands of a 60 km² study area located in Northern Apennines (Alessandria province, Piedmont Region, Italy).
